# Z1456467176 alleviates gouty arthritis by allosterically modulating P2X7R to inhibit NLRP3 inflammasome activation

**DOI:** 10.3389/fphar.2022.979939

**Published:** 2022-08-16

**Authors:** Xiaoling Li, Yiming Liu, Chengyu Luo, Jinhui Tao

**Affiliations:** Department of Rheumatology and Immunology, The First Affiliated Hospital of USTC, Division of Life Sciences and Medicine, University of Science and Technology of China, Hefei, China

**Keywords:** gout, P2X7 receptor, allosteric regulation, nod-like receptor protein 3, interleukin-1beta

## Abstract

NLRP3 inflammasome activation is a central process in initiating gout flares. The unique conformational rearrangement of the P2X7 receptor (P2X7R) upon ATP binding is critical for the activation of the NLRP3 inflammasome. However, studies on allosteric modulation of P2X7R in gout treatment are limited. Here, we aimed to investigate the therapeutic implications of targeting P2X7R in gout by designing a P2X7R allosteric inhibitor and validating the inhibitory function on NLRP3 inflammasome activation. Through virtual screening, we identified Z1456467176 (N-{3-[(2-aminoethyl) sulfamoyl] phenyl}-2-methyl-3-[3-(trifluoromethyl) phenyl] propanamide hydrochloride) bound to the drug-binding pocket as a potential antagonist of P2X7R. In functional assays, ATP- or BzATP-induced P2X7R function was assessed *in vitro* in HEK-293T cells overexpressing hP2X7R (dye uptake assay) and macrophages (IL-1β release assay). Z1456467176 exhibited a stable and significant P2X7R inhibitory effect. Importantly, in MSU crystal-induced gout, the presence and involvement of ATP were confirmed. Z1456467176 blocked ATP-induced activation of the NLRP3-caspase-1-IL-1β pathway and exerted promising effects in reducing gouty joint inflammation in rats. In addition, molecular docking and molecular dynamics simulation studies showed that the P27XR protein conformation was remodeled by Z1456467176 binding. Collectively, our results provide a potent P2X7R allosteric inhibitor that facilitates the remission of MSU crystal-induced gout inflammation by inhibiting NLRP3 inflammasome activation, suggesting that allosteric inhibition of P2X7R represents a new direction in gout treatment.

## Introduction

Gout is a common cyclical, recurrent and transient “autoinflammatory” disease in adults, characterized by rapidly developing monoarticular synovitis of peripheral joints, manifesting as joint redness, swelling, pain, and restricted movement (Dalbeth et al., 2019). For the occurrence of gout flares, monosodium urate (MSU) crystal-induced nod-like receptor protein 3 (NLRP3) inflammasome activation is the central link in initiating the inflammatory response (Martinon et al., 2006). However, this is not sufficient to explain the absence of gout flares in individuals with MSU crystal deposition (Carr et al., 2016; Petsch et al., 2016; Zhu et al., 2017). In addition to MSU crystals, it has been shown that metabolic substances in the body, including adenosine triphosphate (ATP), cholesterol, and glucose, contribute to the activation of the NLRP3 inflammasome (Di Virgilio et al., 2017; Hughes and O'Neill, 2018).

Purinergic receptor P2X7 (P2X7R) is an ATP-gated ion channel receptor that regulates NLRP3 inflammasome activation by inducing intracellular and extracellular ion flux (Murakami et al., 2012; Gong et al., 2018). The association between human *P2X7R (hP2X7R)* gene polymorphisms and gout susceptibility was confirmed in our previous study (Tao et al., 2017). In addition, colchicine, a traditional gout therapeutic agent, has been reported to exert anti-inflammatory effects by inhibiting P2X7R activation-induced pore formation (Marques-da-Silva et al., 2011), suggesting that P2X7R would be an attractive therapeutic target for gout. The P2X7R protein is a trimeric complex with each P2X7 subunit consisting of an extracellular ligand-binding region, two transmembrane structural domains (TM), and intracellular N and C termini (Karasawa and Kawate, 2016). As an allosteric protein, a comparison of the structures in the closed and open states of P2X7R shows that the upper body domain remains relatively quiescent (Jiang et al., 2013). However, ATP binding brings about a narrowing of the turrets and a widening of the lower body domain (Karasawa and Kawate, 2016), and P2X7R antagonist binding precludes these constrictions, efficiently blocking receptor activation (Jiang et al., 2013). This conformational rearrangement is required for P2X7R channel opening (Karasawa and Kawate, 2016; McCarthy et al., 2019), which is essential for NLRP3 inflammasome activation. Therefore, allosteric modulation of P2X7R may be an effective strategy to block NLRP3 inflammasome activation for the treatment of gout.

Although the involvement of ATP-P2X7R in the pathogenesis of gout is gradually being recognized, the evaluation of P2X7R for the treatment of gout is still limited. In the present study, we demonstrated that allosteric regulation of P2X7R to inhibit the NLRP3 inflammasome effectively alleviates gout inflammation, providing theoretical and data support for P2X7R as a therapeutic target for gout.

## Materials and methods

### Z1456467176

Docking studies were performed using the Glide module of Schrodinger software to initiate virtual screening of P2X7R antagonists in a commercial compound library (TSBiochem, China). The crystal of PDB number 5u1x was identified as the docking model. A small-molecule Z1456467176 (N-{3-[(2-aminoethyl) sulfamoyl] phenyl}-2-methyl-3-[3-(trifluoromethyl) phenyl] propanamide hydrochloride) was obtained that binds to the drug antagonist pocket. The purity of the compound was verified by mass spectrometry to be over 90% (Supplementary Data).

### Dye uptake assay

HEK-293T cells were maintained in DMEM (Thermo 11965118) supplemented with 10% fetal bovine serum (FBS, HyClone SV30087.03) and incubated at 37°C under a 5% CO_2_ atmosphere for 24 h (2 × 10^5^ cells/ml). Transfection was performed with a lentivirus overexpressing hP2X7R (Hanheng Biotechnology Co (China)) or empty lentivirus in the presence of polybrene. On the following day, Z1456467176 (10 μM) or equivalent amounts of DMSO were incubated with HEK-293T cells overexpressing hP2X7R for 30 min. Cells were suspended in 0.5 ml of HEPES buffered medium followed by the addition of 25 μM ethidium bromide (EB, Sigma 1,239-45-8) and 1 mM ATP (Sigma A6559). Cells were collected using a CytoFLEX flow cytometer, counting 1,000 cells each time. The average fluorescence intensity of each sample was read using CytExpert. The IC_50_ value of Z1456467176 (concentrations ranging from 1 nM to 100 μM) was obtained from concentration-response curves for the EB uptake assay (Supplementary Figure S1).

### Cell culture and stimulation

THP-1 cells (2 × 10^5^ cells/ml) were cultured in RPMI 1640 medium (HyClone AG29775810) containing 10% FBS and stimulated overnight with 100 ng/ml phorbol 12-myristate 13-acetate (PMA, Sigma P1585) to obtain THP-1-derived macrophages. Bone marrow cells were isolated and collected from bilateral femurs and tibiae of 6-week-old C57BL/6J mice. Mouse bone marrow-derived macrophages (BMDMs) were obtained by culturing for 1 week in DMEM (2 × 10^6^ cells/ml) supplemented with 10% FBS, 20% L929 cell supernatant, and penicillin/streptomycin. Peripheral blood mononuclear cells (PBMCs) from stable gout patients were isolated with lymphocyte isolate and erythrocyte lysate (Haoyang LTS1077, NH4CL 2009) and inoculated in RPMI 1640 medium (2 × 10^6^ cells/ml) and cultured overnight. The supernatant was removed the next day to obtain crude extracted macrophages.

The above cells were then activated with 50 ng/ml LPS (Invitrogen LPS-EK) for 3 h. Furthermore, Z1456467176 at different concentrations was incubated for 30 min before 100 μM BzATP (Sigma B6396) treatment for 15–20 min 10 μM pyroptosis inhibitor (VX-765, MCE HY-13205) was incubated for 30 min before 100 μg/ml MSU crystal treatment for 4 h. MSU crystals were obtained by dissolving uric acid (2 g, Sigma U0881) in 500 ml of double-distilled water containing NaOH (0.01 M) at 70°C, and the pH was maintained at 8.9. The solution was filtered through a 0.22 μM filter and stored overnight at room temperature with continuous stirring. After centrifugation, the supernatant was discarded, and the precipitate was obtained and dried. The crystals were autoclaved and resuspended in sterile PBS (Chen et al., 2019).

### ELISA

ELISA was performed to measure IL-1β, IL-6, and TNF-α levels in cell culture supernatants and IL-1β levels in rat serum according to the manufacturer’s instructions (NOVUS Biological VAL101, VAL102, VAL105; Multi Sciences A301BH10152). Rat liver and kidney functions were detected by Jian Cheng C0009-2-1, C0010-2-1, C011-2-1, and C013-2-1 kits (China).

### Quantitative PCR

qPCR was performed to detect the gene expression of *IL-1β* and *Actβ* in the peripheral whole blood cells of rats. Samples were processed with a total RNA extraction kit (Solarbio R4161-02) and a cDNA synthesis kit (BIOER BSB40M1) according to the manufacturer’s instructions. qPCR was conducted using SYBR Green PCR Master Mix (Muma A4004M). The primer sequences for the rat *IL-1β* gene were F-seq (5′-3′): TGG​CAA​CTG​TCC​CTG​AAC​TC and R-seq (5′-3′): AAG​GGC​TTG​GAA​GCA​ATC​CTT​A. The primer sequences for the rat *Actβ* gene were F-seq (5′-3′): ACCCGCCACCAGTTCGC and R-seq (5′-3′): CACGATGGAGGGGAAGACG.

### Western blotting

Western blotting was performed to detect protein levels in PBMC lysates from gout patients and in joint grinds from rat gout models. Segmented constant voltage was chosen for electrophoresis, and the concentrated gel was run at a constant voltage of 80 V. After running out of the concentrated gel, the voltage was increased to 120 V until bromophenol blue reached the bottom of the gel. The membrane was rotated at a constant flow of 200 mA in an ice bath and then blocked with 5% skim milk powder for 1 h. The membrane was incubated overnight at 4°C with the following primary antibodies: IL-1β (CST D3A3Z, 1:1,000), caspase-1 (CST D7F10, 1:1,000), NLRP3 (CST D4D8T, 1:1,000), N-GSDMD (Abcam Ab215203, 1:1,000) and β-actin (CST 8H10D10, 1:1,000). HRP, goat anti-rabbit IgG, and goat anti-mouse IgG (Abbkine A21020, A21010, 1:5,000) were incubated for 1 h at room temperature and then developed.

### Monosodium urate crystal-induced gouty arthritis in rats

Sprague–Dawley (SD) male rats weighing 200 g were purchased from the Laboratory Animal Center of the First Hospital of USTC and housed in a clean environment free of pathogens. A rat model of gout was established according to the classical modeling method (Coderre method (Coderre and Wall, 1987)) and randomly divided into four groups. Each group was treated as follows: 1) Rats in the control group were injected with 100 μl of sterile saline in the right ankle joint cavity. 2) Rats in the MSU group were injected with 100 μl of MSU crystal suspension (0.1 g/ml) in the right ankle joint cavity. 3) Rats in the MSU + ATP group were injected intraperitoneally with 500 μl of ATP solution (10 mM; Solarbio A8270) 30 min before intra-articular injection of MSU crystal suspension. 4) Rats in the MSU + Z1456467176 group were injected intraperitoneally with 500 μl of Z1456467176 solution (40 mg/kg) 30 min before intra-articular injection of MSU crystal suspension. The clinical inflammatory manifestations of rats were evaluated by measuring the ankle joint circumference and calculating the joint swelling index. Rat ankle joint swelling index = (treated circumference - initial circumference)/initial circumference. Rat ankle joints were obtained from each group and tissue homogenates were prepared according to the manufacturer’s instructions. The activity of myeloperoxidase (MPO, Jiancheng A044-1-1) was assayed as a quantitative assay for tissue neutrophil infiltration.

### LDH and ATP assay

Serum was collected from gout model rats as well as from patients with acute phase gout and normal population. LDH levels were quantified using the LDH Cytotoxicity Assay Kit (Jian Cheng A020-2) according to the manufacturer’s instructions. Patients with acute-phase gout were defined as positive joint redness, swelling, and pressure pain in the metatarsophalangeal or ankle or knee joint; elevated joint skin temperature; history of hyperuricemia or gout; and duration of this joint symptom for 2 days and less. The people in the control group were age- and sex-matched to the gout group and had no local or systemic inflammatory symptoms.

ATP (%) values in cell cultures of PBMCs from rats with MSU crystal-induced gouty arthritis and patients with stable gout stimulated with MSU crystals (100 μg/ml) were determined using a luminescent ATP assay kit (Abcam ab113849) according to the manufacturer’s instructions.

### Magnetic resonance imaging

Rats were anesthetized with intraperitoneal injection of chloral hydrate (0.3 ml/100 g, Maclean C804539) in a gouty arthritis model, and magnetic resonance imaging (MRI) examination was performed after the rats had decreased muscle strength and stabilized respiration. MRI of the ankle joint was performed using a 3.0T MR scanner (GE Discover 750 W) and an eight-channel mouse coil (Medciol MS80-3T) with the following sequence parameters: T2 FLAIR; TE: 101/Ef; TR: 3,800 ms; EC:1/1 50 Hz; slice thickness: 2 mm; FoV = 12*12 cm; matrix size = 256*256.

### Immunohistochemistry

The isolated rat ankles were fixed in tissue fixative (containing 10% neutral formalin) for 24 h, and subsequently placed in decalcification solution (containing formalin and EDTA) with shaking for 4-6 weeks to ensure adequate decalcification. The endpoint of decalcification is indeed considered smooth penetration of the needle through the entire bone tissue. After the washing, dehydration, and decalcification steps, the ankle tissue was fixed in paraffin and sectioned for HE staining. Cell count quantification was performed using ImageJ software (NIH).

### Molecular dynamics analysis and molecular docking

The trimeric conformation of 5u1x was used as the target protein. Molecular docking was performed using AutoDock 4.2 software. The structure of Z1456467176 was downloaded from the database (https://pubchem.ncbi.nlm.nih.gov), and Chem3D16 was applied for structure optimization. Global docking was used, with the docking box wrapping the entire 5u1x protein and other parameters kept as default. Molecular dynamics simulations of 100 ns were performed, and the binding free energy of the protein and ligand was calculated using g_mmpbsa. The conformation with the lowest energy in the molecular docking results was selected as the initial structure for molecular dynamics. Molecular dynamics (MD) simulations were performed using Gromacs 2019.4 software at constant temperature and pressure as well as periodic boundary conditions. The Amber99sb-ildn force field, TIP3P water model, was applied. The force field parameters for small molecules were generated by the acpype.py script in AmberTools. During the MD simulations, the hydrogen bonds involved were constrained using the LINCS algorithm with an integration step of 2 fs. The electrostatic interactions were calculated using the particle‒mesh Ewald (PME) method with the cutoff value set to 1.2 nm. The nonbonded interaction cutoff value was set to 10 Å. The simulation temperature was controlled to 300 K using the V-rescale temperature coupling method, and the NVT and NPT equilibrium simulations were performed at 300 K for 100 ps. Finally, 100 ns of finished MD simulations were performed for the protein and protein-ligand complex systems, and the pure protein system was used as a control group for the experiments. The GROMACS embedded program and PyMOL 2.4 were used to visualize the simulation results.

### Ethics

Our protocols for the collection and processing of animal and human samples were approved by the Animal Ethics Committee (2021-N(A)-041) and Medical Research Ethics Committee(2021 KY No.162) of the First Hospital of the University of Science and Technology of China (Anhui Provincial Hospital), respectively. All persons gave their informed consent before their inclusion in the study.

### Statistical analyses

Data were analyzed using IBM SPSS Statistics for Windows, version 17.0 (IBM). Quantitative data were tested for normality. Independent sample *t* tests and one-way analysis of variance (ANOVA) were used to compare differences between two groups and multiple groups, respectively. Significant differences revealed by one-way ANOVA were validated by Dunnett’s *t* test. Statistical significance was set at *p* < 0.05. All bar graphs were plotted using GraphPad Prism 8.0 software (GraphPad Software).

## Results

### Z1456467176 inhibits ATP-induced P2X7R pore formation

**FIGURE. 1:**
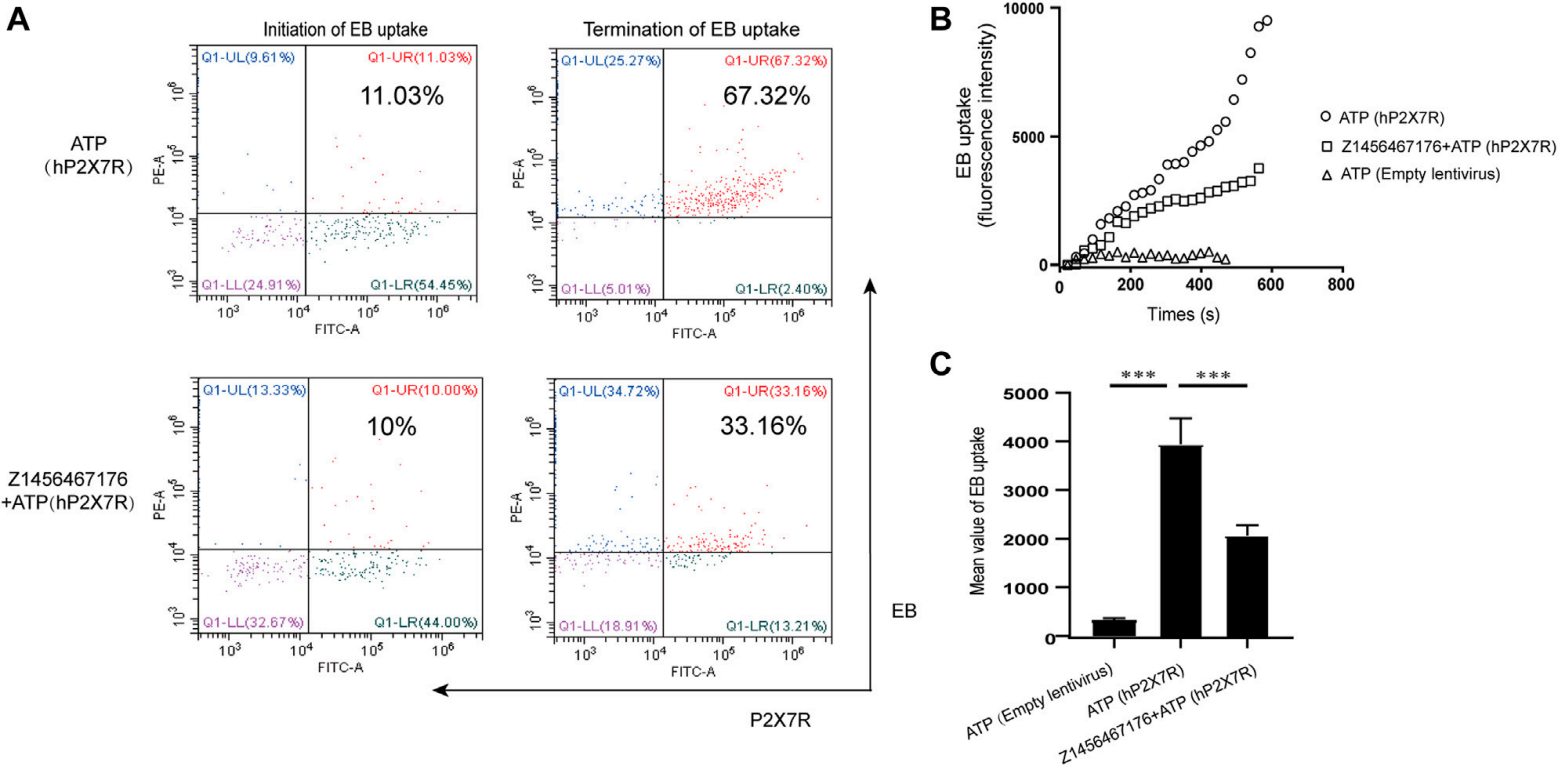
Z1456467176 inhibits ATP-induced P2X7R pore formation. **(A)** Representative flow diagram of EB uptake by HEK-293T cells overexpressing hP2X7R in response to ATP. The red Q1-UR region reflects double-positive staining for EB and hP2X7R. FACS analysis of the intracellular fluorescence intensity **(B)** and mean value analysis of quantified intracellular fluorescence intensity **(C)**. Data are presented as the means ± S.E.M.s. Statistics were analyzed using Dunnett’s *t*-test. ***p < 0.001.

P2X7R is an ATP-gated channel receptor. Pore formation upon sustained stimulation of ATP allows the influx of extracellular macromolecules. To investigate the inhibitory effect of Z1456467176 on ATP-induced P2X7R pore formation, we constructed a lentivirus overexpressing hP2X7R to transfect HEK-293T cells. ATP-induced uptake of EB by HEK-293T cells was assayed in the presence or absence of Z1456467176. Flow cytometric analysis showed that the uptake of EB by HEK-293T cells overexpressing hP2X7R was significantly increased upon ATP stimulation, whereas Z1456467176 treatment inhibited the uptake of EB by the cells (Figure 1). This finding indicates that Z1456467176 (EB uptake IC_50_: 3.416 μM) was able to inhibit ATP-induced P2X7R pore formation, exhibiting a potential P2X7R inhibitory effect.

### Z1456467176 inhibits BzATP-induced IL-1β secretion in macrophages

**FIGURE. 2:**
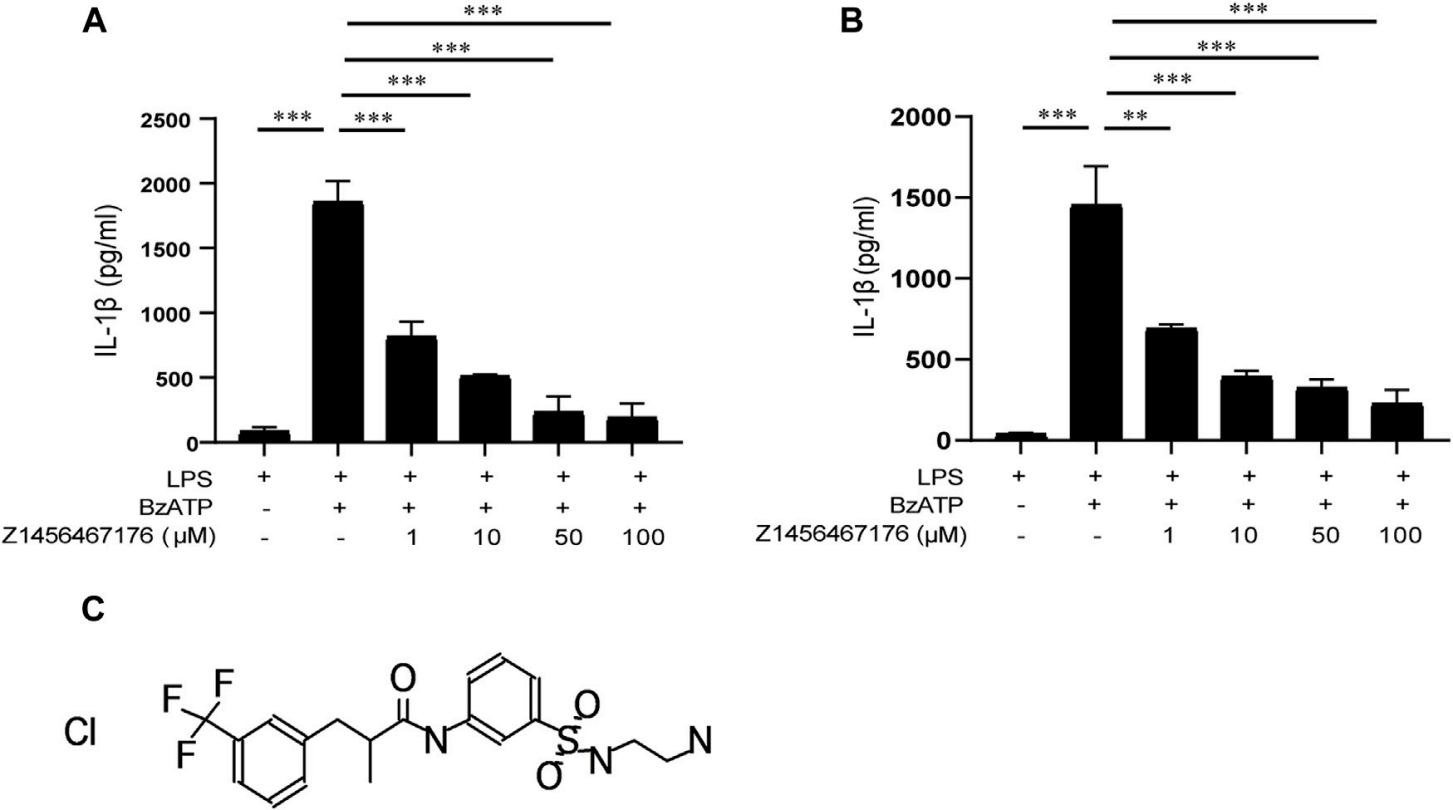
Z1456467176 inhibits BzATP-induced IL-1β secretion in macrophages. ELISA of IL-1β in supernatants from LPS (50 ng/ml)-primed THP-1 cells **(A)** and BMDMs **(B)** treated with various doses of Z1456467176 (1/10/50/100 μM) for 30 min and then stimulated with BzATP (100 μM). **(C)** Chemical structure of Z1456467176. Data are presented as means ± S.E.M. Statistics were analyzed using Dunnett’s *t*-test. *n* = 3. **p < 0.01; ***p < 0.001.

P2X7R has attracted widespread interest as a therapeutic target for gout, as it mediates IL-1β release in response toATP. The release of biologically active IL-1β in gout requires two steps: initiation and activation, a biological process that occurs mainly in macrophages. We next investigated the effect of Z1456467176 on P2X7R activation-induced IL-1β release in an *in vitro* macrophage system including THP-1 cells and BMDMs. BzATP is a synthetic ATP analog that acts only on P2X1R and P2X7R in the P2XR family and exhibits a more potent and specific induction of P2X7R activation than ATP (Gicquel et al., 2017). Thus, BzATP was used to stimulate P2X7R activation in macrophages. As shown in Figure 2, we observed that in LPS-primed THP-1 cells and BMDMs, Z1456467176 was able to inhibit BzATP-induced IL-1β secretion in a dose-dependent manner to varying degrees, suggesting that it functions as a P2X7R antagonist.

With the validation of effectiveness in inhibiting P2X7R pore formation and IL-1β secretion in macrophages of different species *in vitro*, Z1456467176 was identified as a P2X7R antagonist that inhibits ATP- or BzATP-induced P2X7R activation.

### Z1456467176 inhibits P2X7R-NLRP3-IL-1β pathway activation *in vitro*

**FIGURE. 3:**
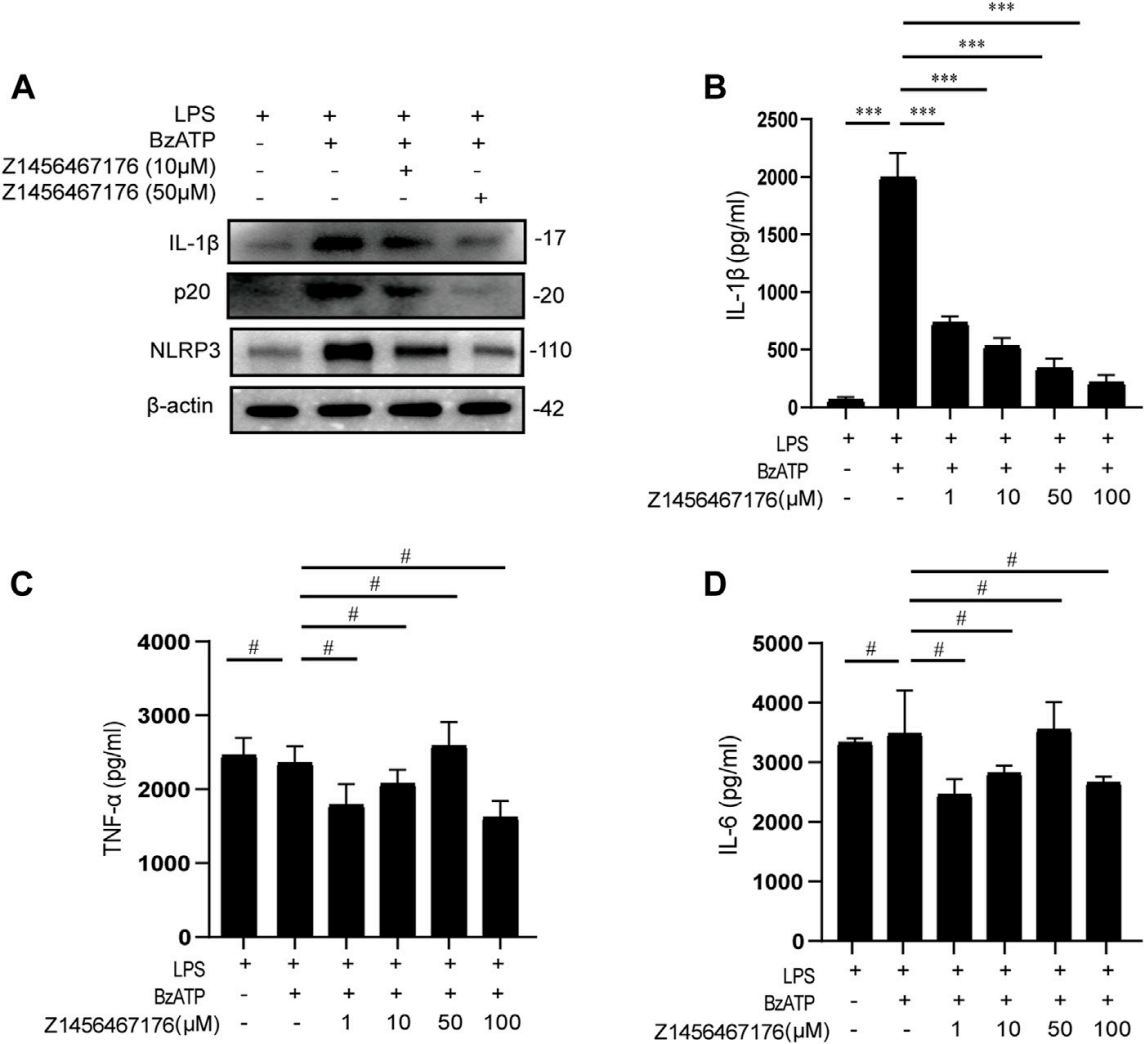
Z1456467176 inhibits the P2X7R-NLRP3-IL-1β pathway *in vitro*. **(A)** WB of NLRP3, caspase-1 (p20), and IL-1β in lysates from LPS-primed PBMCs of gout patients treated with Z1456467176 (10, 50 μM) for 30 min and stimulated with BzATP (100 μM). **(B–D)** ELISA of IL-1β, TNF-α, and IL-6 in culture supernatants from LPS-primed PBMCs of gout patients treated with Z1456467176 for 30 min and stimulated with BzATP. Data are presented as the means ± S.E.M.s. Statistics were analyzed using Dunnett’s *t* test. *n* = 3. ***p < 0.001.

To provide a basis for Z1456467176 in the treatment of gout, we evaluated the inhibitory effect of Z1456467176 on the P2X7R-NLRP3-IL-1β pathway in cultures of PBMCs of gout patients. First, we investigated whether Z1456467176 affected P2X7R-induced NLRP3 inflammasome activation in gout. As expected, western blot results showed that BzATP-induced expression of NLRP3, caspase-1 and IL-1β was inhibited in the presence of Z1456467176 (Figure 3A). Since IL-1β secretion is mainly mediated by activated NLRP3 inflammasome, we then examined inflammasome-dependent IL-1β secretion in culture supernatants of PBMCs from gout patients. The results showed that Z1456467176 inhibited BzATP-induced IL-1β secretion in cell culture supernatants in a dose-dependent manner, whereas NLRP3 inflammasome-independent TNF-α and IL-6 levels were unaffected (Figures 3B–D). In addition, we excluded the effect of Z1456467176 on LPS- primed and nigericin-induced NLRP3 inflammasome activation (Supplementary Figure S2). Taken together, these data suggest that Z1456467176 blocks the NLRP3-IL-1β pathway by inhibiting BzATP-induced P2X7R activation in gout patients *in vitro*.

### Z1456467176 alleviates gouty arthritis in rats

**FIGURE. 4:**
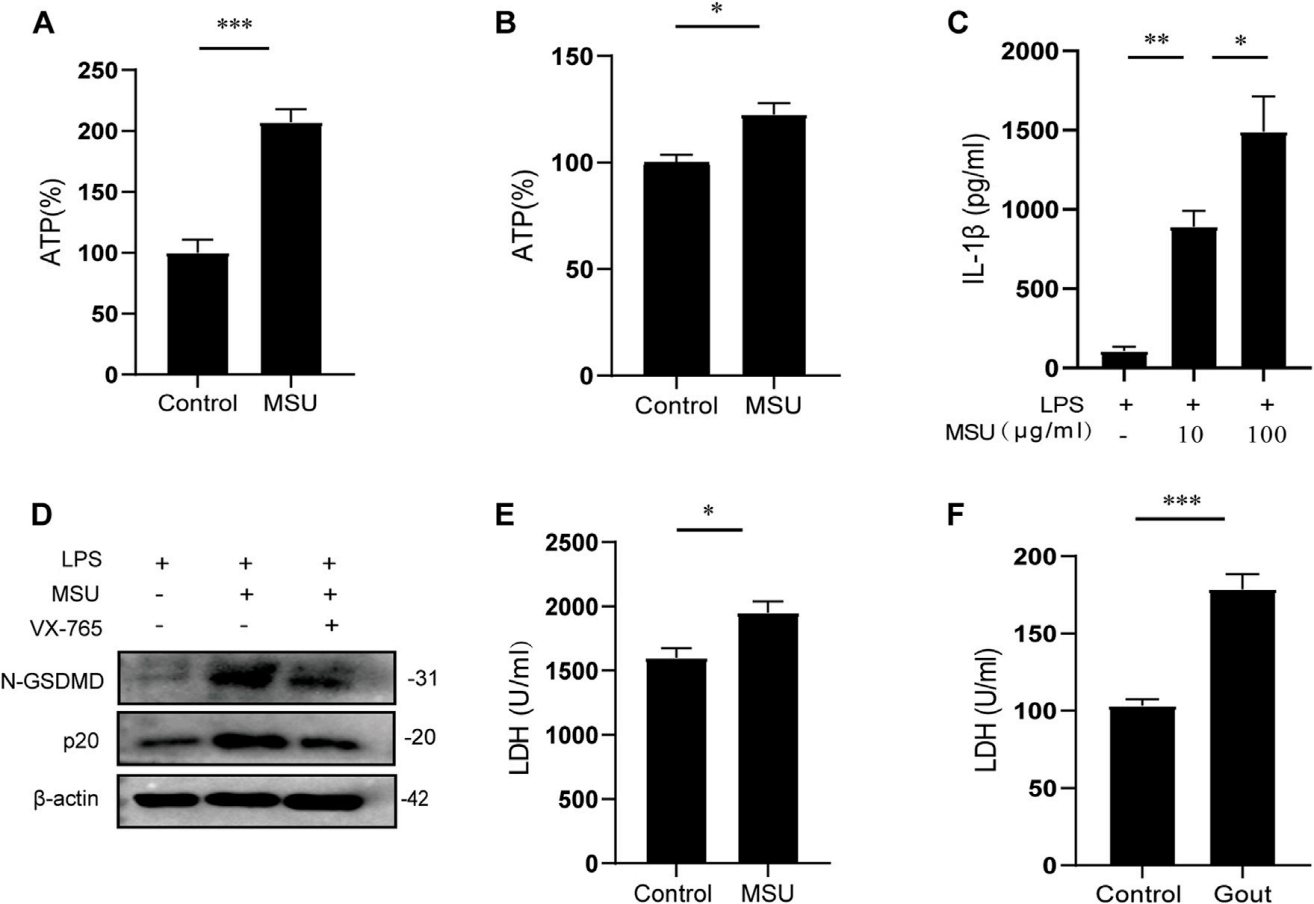
MSU crystal stimulation induces ATP production and release. ATP (%) of cell cultures of PBMCs from MSU crystal (0.1 g/ml)-induced gouty arthritis rats **(A)** and LPS (50 ng/ml)-initiated, MSU crystal (100 μg/ml)-stimulated stable gout patients **(B)**. Control groups were treated with equal amounts of sterile saline or PBS. The ATP (%) values of **(A)** and **(B)** are expressed as a percentage of the MSU group relative to the control group. **(C)** ELISA of IL-1β in culture supernatants from LPS-primed PBMCs of gout patients treated with MSU (10/100 μg/ml) for 4 h **(D)** WB of N-GSDMD and p20 expression in lysates from LPS-primed PBMCs of gout patients treated with VX-765 (pyroptosis inhibitor; 10 μM) for 30 min and then stimulated with MSU (100 μg/ml). LDH levels in the serum of MSU crystal-induced gout rats **(E)** and in acute-phase gout patients **(F)**. Data are presented as the means ± S.E.M.s. Statistics were analyzed using the independent samples *t* test or Dunnett’s *t* test. **(A,B)** *n* = 3-4; **(C)** *n* = 4; **(E)** *n* = 5; **(F)** *n* = 19. *p < 0.05; **p < 0.01; ***p < 0.001.

To investigate the therapeutic effect of Z1456467176 on gout *in vivo*, we first evaluated inflammatory manifestations in a traditional gout model rat. In this model, MSU crystals were injected into the ankle joint cavity of rats to construct gouty arthritis (Coderre and Wall, 1987). Interestingly, ATP did not seem to be involved in this process. However, elevated levels of ATP were detected in the cell cultures of PBMCs from MSU crystal-induced gout rats (Figure 4A). Similarly, the effect of MSU crystal stimulation on ATP release was also observed in cell cultures of PBMCs from gout patients (Figure 4B). These data suggest that MSU crystal stimulation promotes ATP production in gout.

Pyroptosis-induced cell membrane rupture is the main mechanism for the rapid release of cellular contents. To determine whether MSU crystal-induced ATP is released by pyroptosis, the levels of pyroptotic markers in gout were tested. The results showed that extracellular IL-1β and LDH release were increased and intracellular caspase-1 and N-GSDMD expressions were up-regulated after MSU crystal stimulation (Figures 4C–F). Collectively, these data suggest that MSU crystal stimulation promotes ATP production, which is released extracellularly through pyroptosis to function as a signaling molecule, confirming the involvement of ATP signaling in MSU crystal-induced gout pathogenesis.

**FIGURE. 5:**
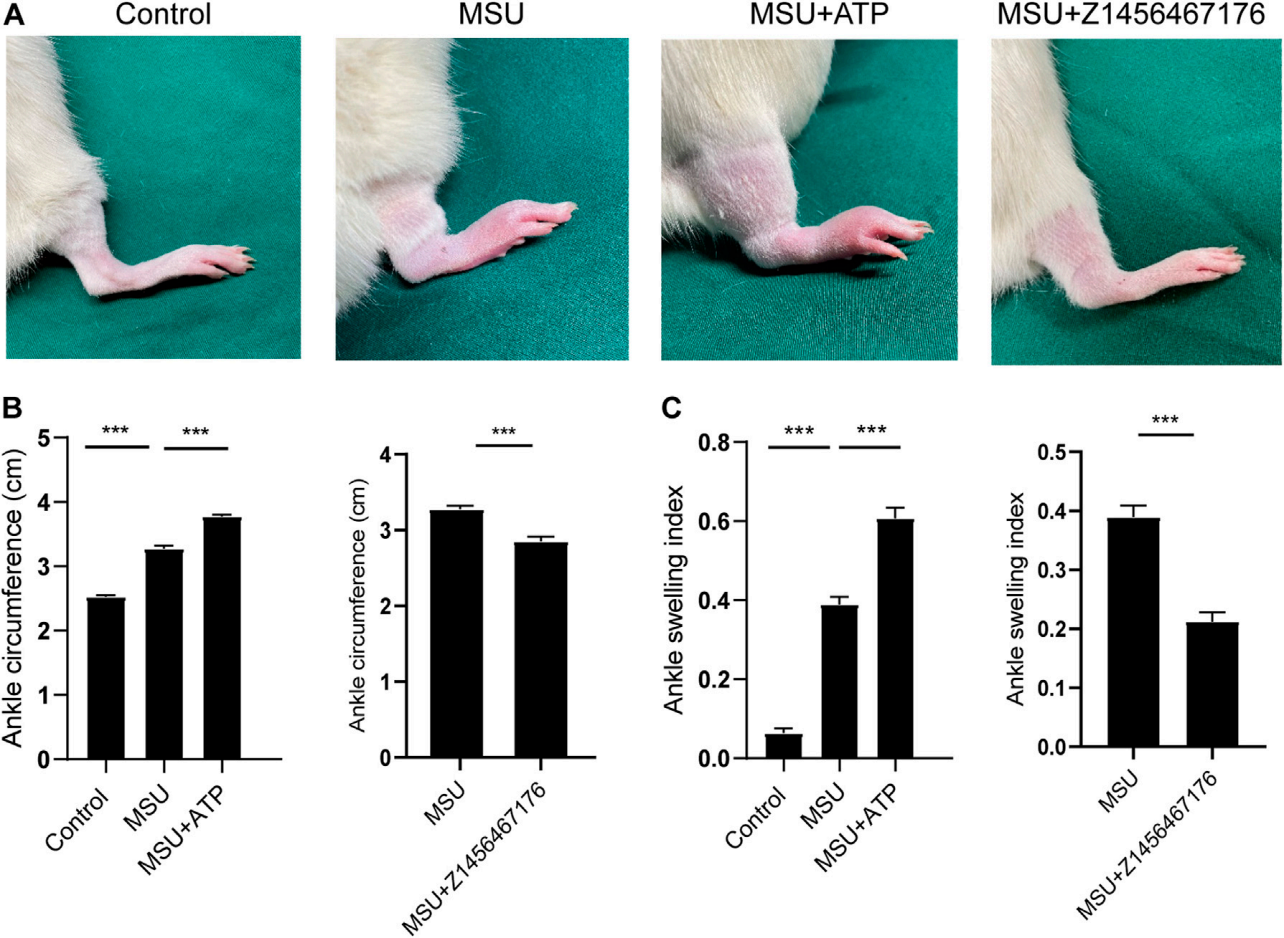
Z1456467176 alleviates gouty arthritis in rats. The rat model of gout was divided into four groups: the control group, MSU group, MSU + ATP group, and MSU + Z1456467176 group. **(A)** Representative diagram of the right ankle joint of rats. **(B,C)** Ankle joint circumference and joint swelling index of the four groups of rats. Data are presented as the means ± S.E.M.s. Statistics were analyzed using the independent samples *t* test or Dunnett’s *t* test. *n* = 4. ***p < 0.001.

**FIGURE. 6:**
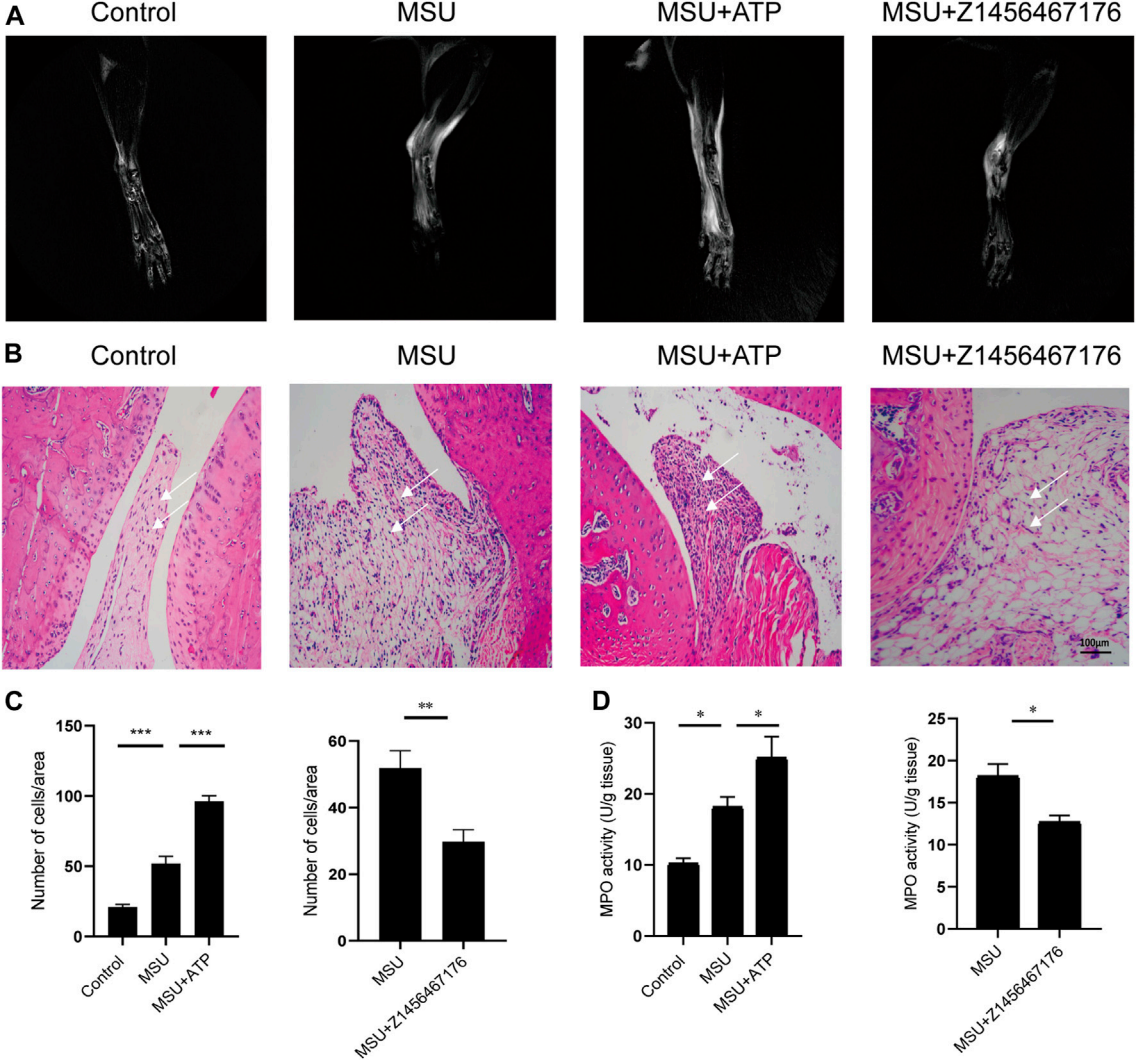
Z1456467176 alleviates gouty arthritis in rats. **(A)** Representative MRI images of the right ankle joint of rats. The white portion reflects the degree of inflammatory edema. **(B)** Representative images of pathological H&E staining of the right ankle joint of rats (×200). The white arrow points to inflammatory cell infiltration in the synovial tissue of the ankle joint. **(C)** Equal-sized areas were taken from each group in **(B)** for quantification of inflammatory cells. **(D)** MPO activity of ankle joint homogenates. Data are presented as the means ± S.E.M.s. Statistics were analyzed using the independent samples *t* test or Dunnett’s *t* test. *p < 0.05; **p < 0.01; ***p < 0.001.

Based on this, we established a rat model of MSU crystal-induced gout to validate the therapeutic effect of Z1456467176 on ATP-P2X7R-associated gout. As expected, in the MSU crystal-induced gout rat model, ATP treatment exacerbated the inflammatory manifestations of arthritis, while intraperitoneal injection of Z1456467176 attenuated the symptoms (Figure 5). Similar to the clinical manifestations, ankle MRI showed that ATP exacerbated MSU crystal-induced inflammation in the soft tissues of the rat ankle joint, while Z1456467176 attenuated it (Figure 6A). In addition, histopathology of the ankle joint showed increased inflammatory cell infiltration in the synovial tissue of the ankle joint of rats in the MSU group compared to the control group. ATP injection exacerbated the MSU crystal-induced inflammatory cell infiltration, while Z1456467176 treatment attenuated it (Figures 6B–D). These results suggest that Z1456467176 effectively relieves MSU-induced gouty joint inflammation.

### Z1456467176 inhibits NLRP3 inflammasome activation in rat model of gout

**FIGURE. 7:**
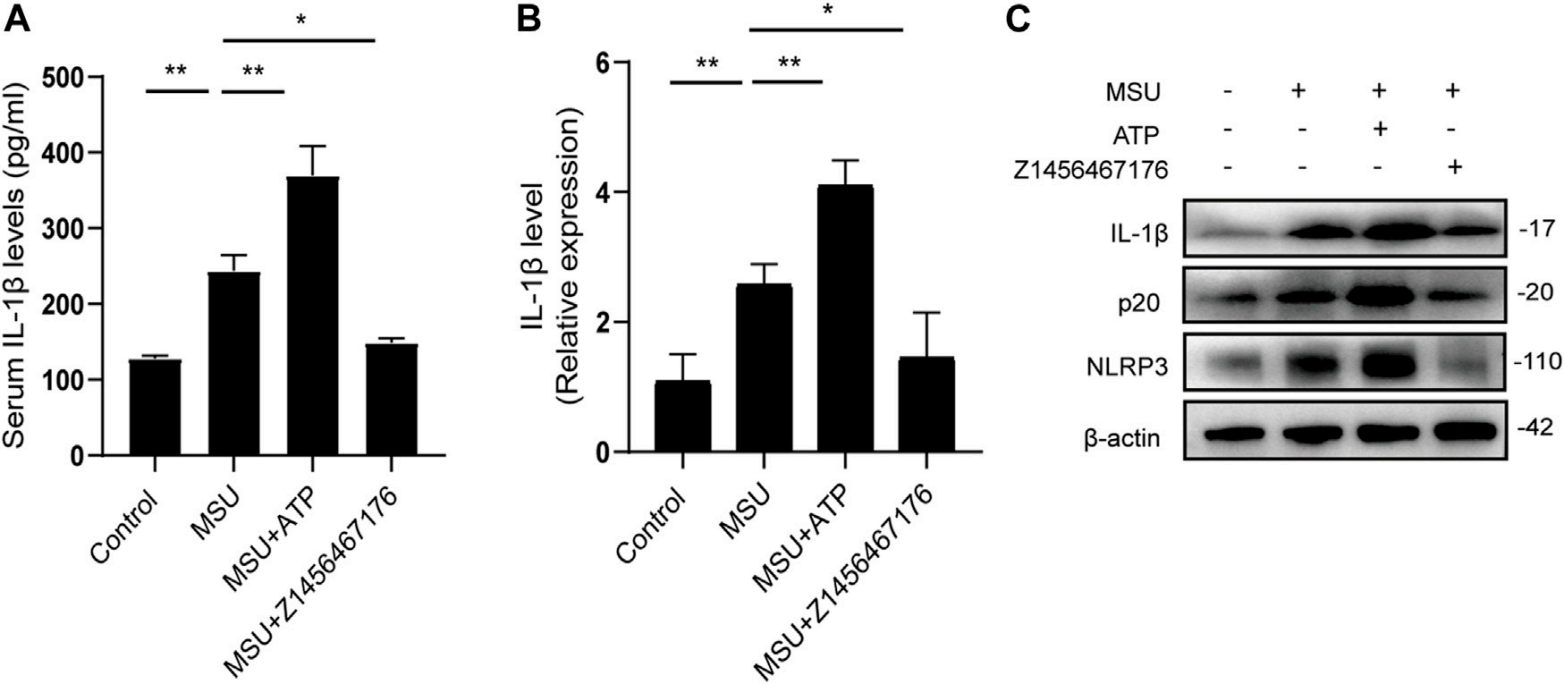
Z1456467176 inhibits NLRP3 inflammasome activation in rat model of gout. **(A)** ELISA of serum IL-1β levels in the four groups. **(B)** qPCR of *IL-1β* gene levels in the peripheral whole blood cells of rats in each group. **(C)** WB of NLRP3, p20, and IL-1β expression in the joints and surrounding synovial tissues of rats in each group. Data are presented as the means ± S.E.M.s. Statistics were analyzed using Dunnett’s *t* test. *n* = 4. *p < 0.05; **p < 0.01.

To further elucidate the mechanism by which Z1456467176 alleviates gout inflammation, we examined the expression of the NLRP3 inflammasome pathway in rats. Consistent with the clinical presentation, ATP treatment resulted in an upregulation of IL-1β levels following MSU crystal stimulation in rats, which was inhibited by Z1456467176 (Figures 7A,B). Western blot results of rat joints showed that NLRP3, caspase-1, and IL-1β expression were upregulated by MSU crystal stimulation. Intraperitoneal injection of ATP exacerbated and Z1456467176 inhibited MSU crystal-induced activation of the NLRP3 inflammasome pathway (Figure 7C). Based on the release of extracellular ATP after MSU crystal stimulation, these data suggest that Z1456467176 effectively alleviates the symptoms of gouty arthritis by acting on P2X7R to inhibit ATP-induced activation of the NLRP3 inflammasome.

To further evaluate the safety of Z1456467176 treatment *in vivo*, we next examined the liver and kidney function levels of rats in each group. The results showed no significant impairment of liver and kidney function in rats treated with Z1456467176 compared to other groups (Table 1).

**TABLE 1:** Biochemical parameters of rats in gout model.

Biochemical index	Control group	MSU group	MSU + ATP group	MSU + Z1456467176 group	*p* Value
AST (U/L)	15.52 ± 1.89	24.41 ± 7.50	17.79 ± 7.21	20.42 ± 9.07	>0.05
ALT (U/L)	19.99 ± 6.34	32.86 ± 10.39	26.47 ± 10.28	28.81 ± 6.48	>0.05
Bun (mmol/L)	1.40 ± 0.17	1.26 ± 0.44	1.33 ± 0.22	2.16 ± 1.69	>0.05
Cr (µmol/L)	28.33 ± 10.07	34.86 ± 29.03	21.2 ± 9.99	34.35 ± 13.86	>0.05

*p* value represents the comparison of MSU + Z1456467176 group and other groups in each index.

AST, aspartate transaminase; ALT, alanine transaminase; Bun, blood urea nitrogen; Cr, creatinine.

### The binding of Z1456467176 rearranges the P2X7R protein conformation

**FIGURE. 8:**
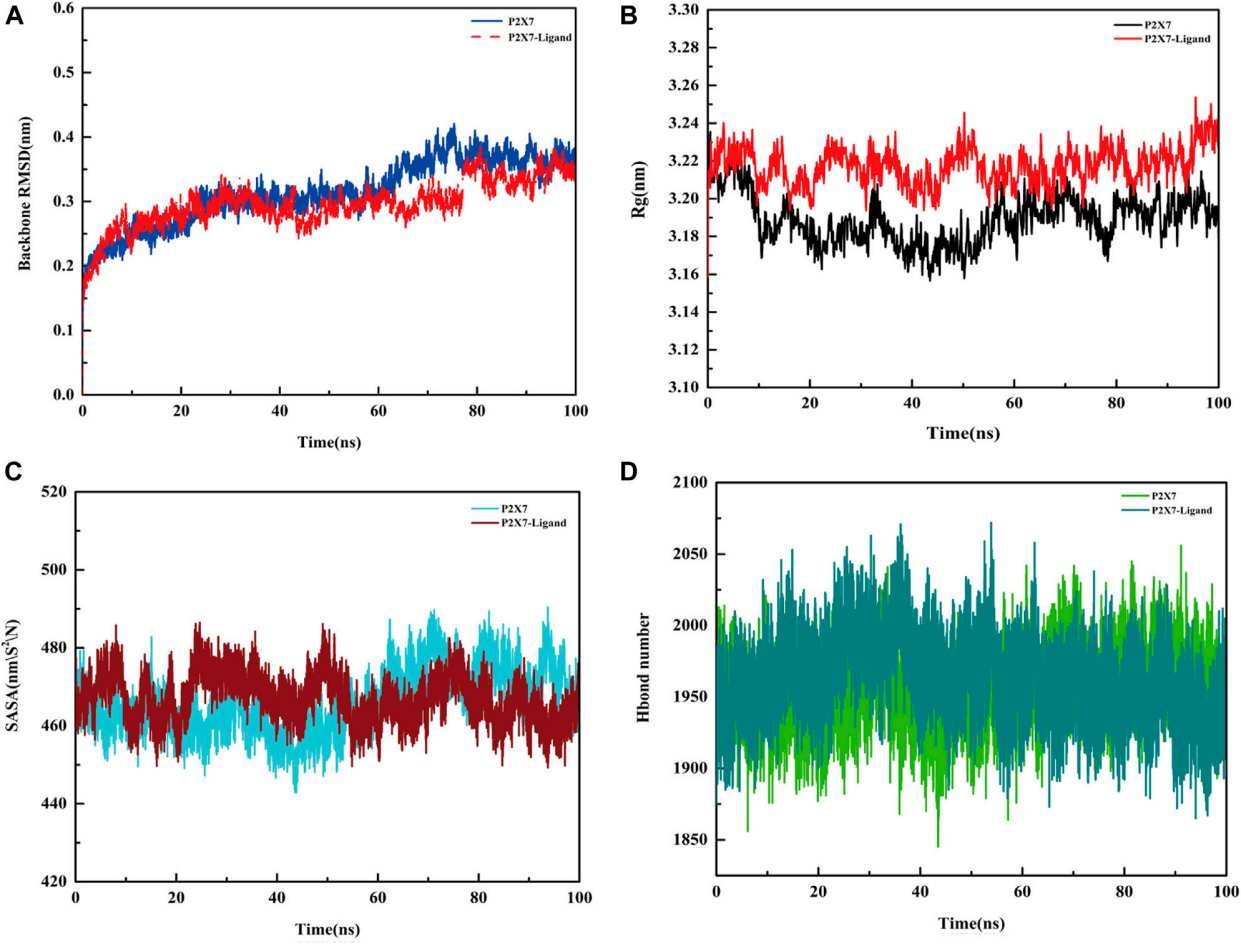
Molecular dynamics simulation of the P2X7R protein and protein‒ligand. **(A)** Variation in RMSD values of the protein (P2X7R, blue line) and protein‒ligand (Z1456467176, red line) systems with time. **(B)** Rg values of the protein (black line) and protein‒ligand (red line) systems with time. **(C)** SASA area of the protein (blue line) and protein‒ligand (red line) systems over time. **(D)** The hydrogen bond formed between the protein (light green line), protein‒ligand (dark green line) system and water.

Molecular dynamics simulation was performed to analyze the binding of the protein (P2X7R)‒ligand (Z1456467176), providing data on receptor and ligand affinity. During the 100 ns kinetic simulations, the RMSD values of the protein‒ligand systems were kept in equilibrium, with mean values of 0.3664 ± 0.0137 nm and 0.3413 ± 0.016 nm, respectively, suggesting that the systems reached stability (Figure 8A). The radius of gyration (Rg) values of the protein‒ligand system were larger than those of the pure protein system throughout the simulation time (Figure 8B), suggesting that the system swelled due to local conformational changes after ligand binding. The solvent accessible surface area (SASA) of the protein after ligand binding was reduced in the 80-100 ns equilibrium period compared to the protein, 463.3 ± 4.7 and 472.4 ± 5.9, respectively, probably due to the reduced contact area of hydrophobic amino acid residues in the ligand-binding region with the solvent (Figure 8C). During the equilibrium period, the mean values of hydrogen bond formation between protein and protein‒ligand were 1968 ± 23.5 and 1947 ± 25.5, respectively, indicating the presence of more hydrogen bonds between water molecules and protein (Figure 8D).

**FIGURE. 9:**
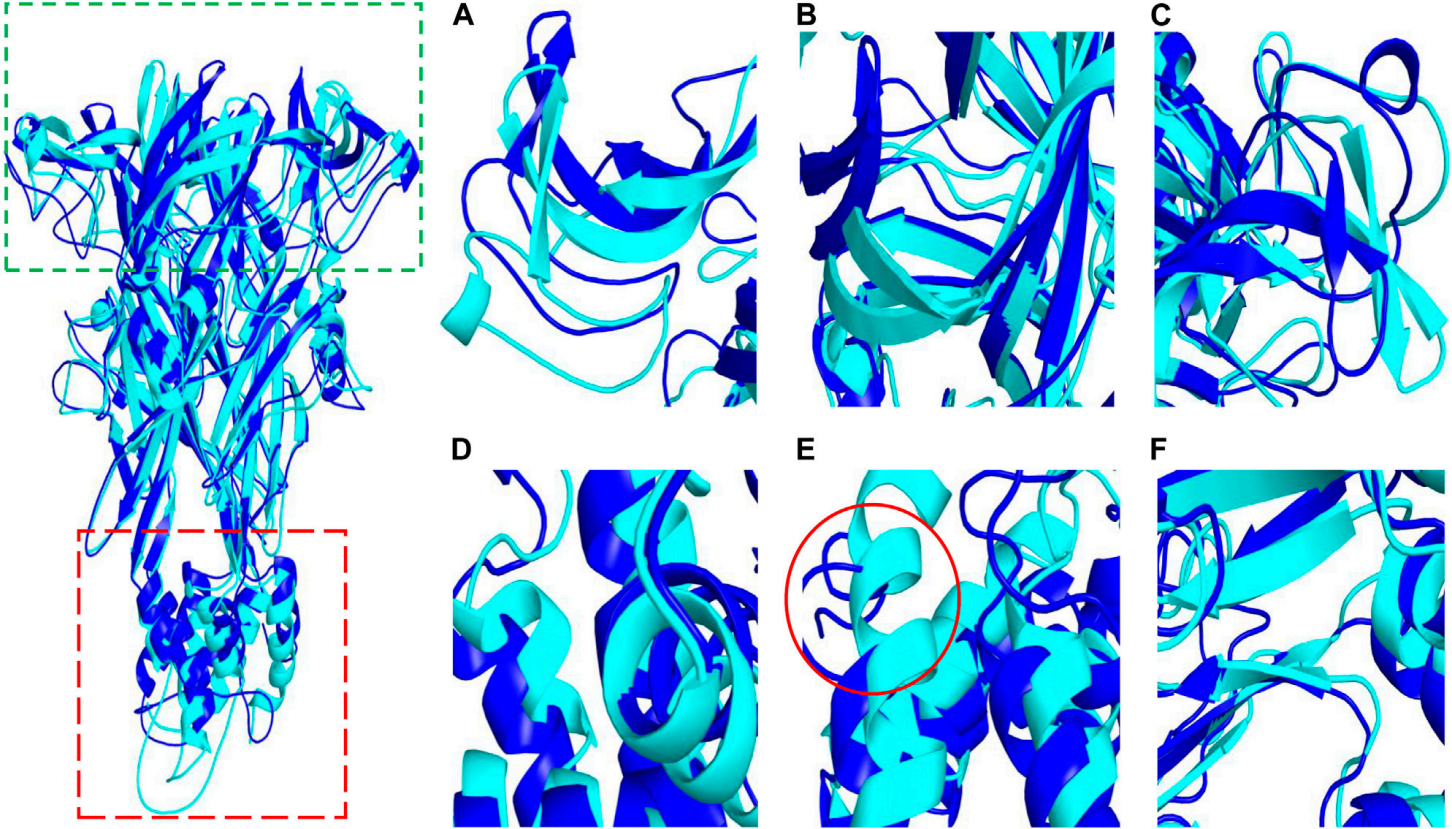
The conformational difference between P2X7R and P2X7R-ligand. The diagram on the left shows conformational differences between the pure protein (P2X7R, blue) and the protein‒ligand (Z1456467176, cyan). The green and red boxes indicate the approximate locations of the domains shown in **(A–C)** and **(D–F)**, respectively. **(A–C)** The conformation of the open region at the upper end of P2X7R. **(D–F)** The conformation of the helical region of P2X7R.

To determine whether Z1456467176 binding resulted in structural rearrangement of the P2X7R protein, we compared the secondary structures of the P2X7R protein and protein‒ligand. The results showed that the open region at the upper end and the TM region at the lower end of P2X7R expanded outwards after ligand binding compared with the pure protein, leading to a change in secondary structure (Figure 9), which was consistent with the change in Rg value. Specifically, Figure 9E shows the conformational transition of the secondary structure after protein‒ligand binding from α-helix to loop. This indicated that the binding of Z1456467176 significantly changed the conformation of the P2X7R protein.

On this basis, we further analyzed the protein‒ligand binding data. The total binding free energy of the protein‒ligand is -60.493 kJ/mol, of which the van der Waal energy is -196.508 kJ/mol; the electrostatic energy is -56.783 kJ/mol; the polar solvation energy is 214.035 kJ/mol; and the SASA energy is -21.236 kJ/mol. The binding free energy of the two shows that the van der Waals force and the electrostatic force are dominant, which is beneficial to the combination. The contribution of amino acid residues at different positions to the binding free energy was obtained by decomposing the binding free energy (Supplementary Table S1).

**FIGURE. 10:**
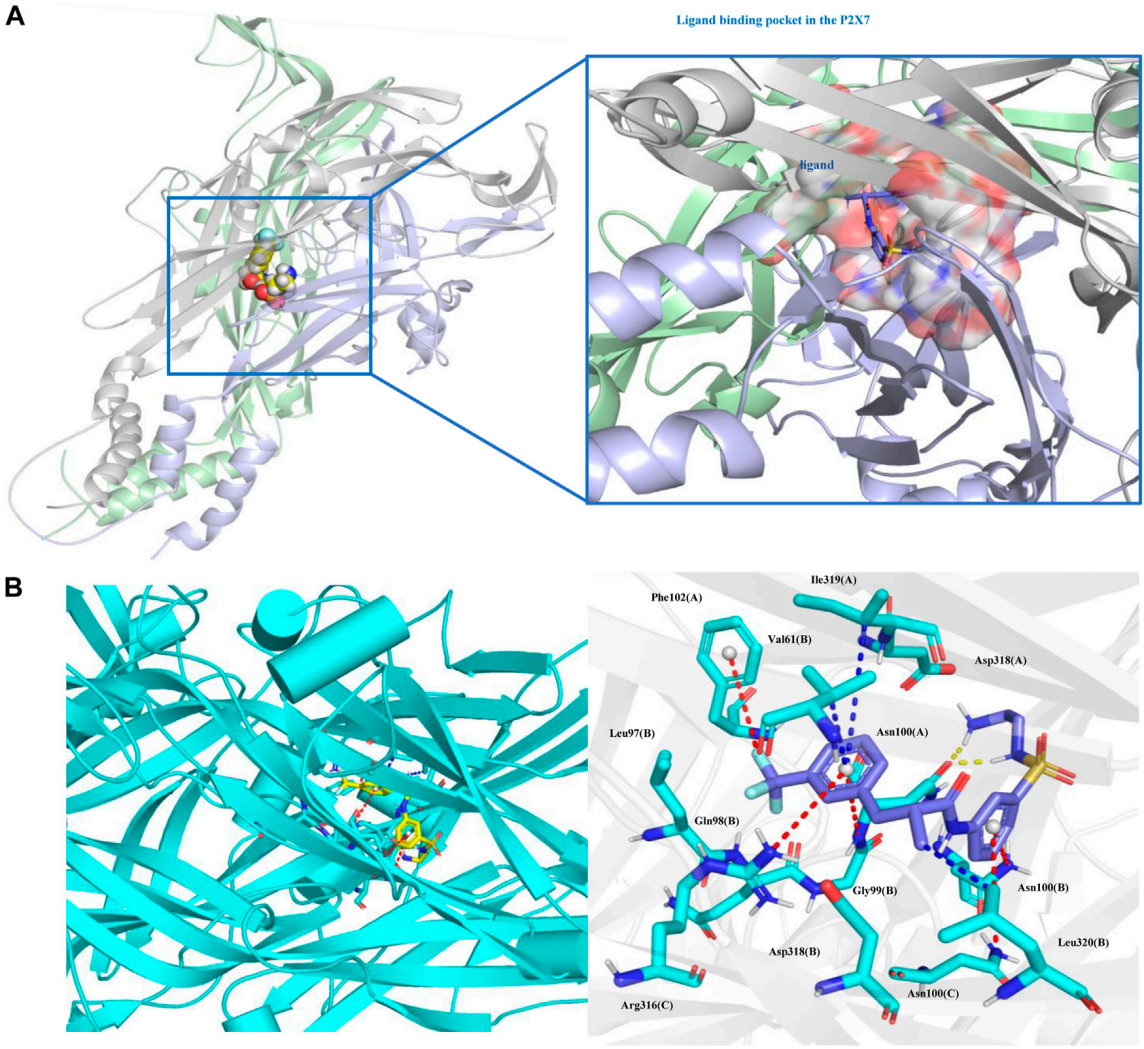
Protein‒ligand binding pattern. **(A)** Protein‒ligand binding site. **(B)** Interacting amino acid residues and interaction force; the red dotted line represents the electrostatic force, the yellow dotted line represents the hydrogen bond, and the blue dotted line represents the hydrophobic force. Ligand (Z1456467176, blue) and P2X7R residues (green) are represented as stick models.

Protein‒ligand binding pattern analysis showed that the pi-cation electrostatic interaction exists between the amino groups of Arg316 (C), Asn100 (A), Asn100 (B), Asn100 (C) and the ligand, and the interaction distance is 5.0 Å, 4.7 Å, 4.8 Å, 5.5 Å. In addition, there is an pi-anion electrostatic interaction between the F atom of the ligand and Phe102 (A) with a distance of 5.2 Å. Pi-alkyl hydrophobic interactions exist between Val61 (B), Ile319 (A), Leu320 (B) and the ligand, with distances of 4.0 Å, 6.0 Å, and 3.6 Å, respectively. Hydrogen bonds are formed between Asn100 (A) and the ligand at distances of 1.8 Å and 2.0 Å, respectively (Figure 10).

## Discussion

In this study, we identified a potent P2X7R antagonist through virtual screening and functional validation. In *vivo and in vitro* studies, Z1456467176 inhibited ATP- or BzATP-induced P2X7R activation to block the NLRP3-caspase-1-IL-1β pathway, alleviating the clinical manifestations of gouty arthritis and confirming P2X7R as an effective therapeutic target. Importantly, we herein found that the binding of Z1456467176 induces a conformational change in the P2X7R protein, suggesting that allosteric inhibition of P2X7R is a promising strategy for gout treatment.

In the past, several competitive and noncompetitive antagonists against P2X7R have been developed. Oxidized ATP is an irreversible competitive P2X7R antagonist but simultaneously exhibits nonspecific modulation of NF-κB signaling pathway expression (Di Virgilio, 2003). Notably, our study showed that Z1456467176 specifically inhibited P2X7R-mediated IL-1β release without affecting IL-6 and TNF-α expression. BBG is a conventional P2X7R antagonist but also affects P2X4R (North, 2002), which is functionally similar to P2X7R (Kanellopoulos et al., 2021), implying a nonselective inhibitory function on P2XRs. Despite the functional similarity, the two receptors are structurally different, with the drug-binding pocket of P2X4R being narrower than that of P2X7R (Sluyter, 2017). Therefore, the antagonists screened by molecular docking based on the P2X7R drug-binding pocket theoretically have higher specificity. In addition, the selectivity of BBG for hP2X7R is lower than that for rats, leading to a limited clinical application (Jiang et al., 2000). KN-62 is an isoquinoline derivative that inhibits human and mouse P2X7R activation, but evidence of inhibition in rats is lacking (Humphreys et al., 1998). Furthermore, KN-62 acts as a complete antagonist to ATP stimulation yet exerts a partial antagonistic effect on the stimulation of the P2X7R-specific agonist BzATP (Humphreys et al., 1998). In the present study, we determined that Z1456467176 responded to different species and exerted effective inhibition of both ATP- and BzATP-induced P2X7R activation.

Currently, we have witnessed the clinical application of several P2X7R antagonists. However, the expected therapeutic effect has not been achieved in the treatment of inflammation-associated diseases (Keystone et al., 2012; Stock et al., 2012; Eser et al., 2015), suggesting that a better understanding of the mechanisms involved in P2X7R-mediated disease pathogenesis is warranted. Gout is characterized by an inflammatory response caused by the deposition of MSU crystals in the joints, cartilage, and surrounding synovial tissue. Gout flares are usually accompanied by triggers including exercise, alcohol intake, overeating, and late nights. Collating these triggers we found that they all cause fluctuations in ATP levels in the body (Lowell and Spiegelman, 2000; Sauer et al., 2000; Mashimo et al., 2015; Alghannam et al., 2021), which points to the possibility that ATP is an important predisposing factor for the development of gout. In this study, we demonstrate that stimulation of MSU crystals induces an increase in ATP production, which is released extracellularly through pyroptosis. Extracellular ATP is a signaling molecule that acts on P2X7R to activate the NLRP3-caspase-1-IL-1β signaling pathway, aggravating MSU crystal-induced gout pathogenesis. Therefore, these results suggest that ATP-P2X7R is involved in the pathogenesis of gout, which is corroborated by a study of *P2X7R* gene polymorphisms and gout susceptibility (Gong and Chen, 2015; Tao et al., 2017). Functionally enhanced *P2X7R* gene polymorphisms in gout patients imply a more sensitive ATP response in these populations, as evidenced by the rapid opening function of P2X7R channels and the massive release of IL-1β (Sorge et al., 2012; Jindrichova et al., 2015). As illustrated by Jiang (Jiang et al., 2013), NS-SNPs characterization of specific residues in P2X7R reveals unique molecular mechanisms that determine differences in P2X7R function, providing new insights into disease mechanisms. P2X7R is a trimeric protein and the conformational changes induced by ATP binding contribute to channel formation (Karasawa and Kawate, 2016). Therefore, screening for allosteric inhibitors that block this process from occurring is a promising direction for gout. The results of molecular docking and kinetic analyses show a greater structural difference in the secondary structure after protein-Z1456467176 binding compared to the pure protein system, exhibiting an outward expansion of the open region at the upper end of P2X7R and the TM region at the lower end. Notably, the closure of the turret located at the upper end of the protein during P2X7R activation is a characteristic conformational change after ATP binding (Karasawa and Kawate, 2016). Thus, structural remodeling leading to the restriction of P2X7R protein response to ATP is responsible for the therapeutic effect of Z1456467176 in gout. A growing understanding of the structure‒function relationship of P2X7R will facilitate continued efforts to optimize the treatment of gout disease.

Z1456467176 achieved significant therapeutic effects in gout rats *in vivo*, although it did not completely inhibit BzATP-induced IL-1β secretion *in vitro*. IL-1β is the main proinflammatory cytokine in initiating gout; however, the IL-1β-mediated inflammatory response is also critical in resolving infections to some extent, contributing to the body’s timely response to danger and self-recovery. In tissue recovery after injury, low levels of IL-1β and TNF-α promote wound healing by increasing growth factor production (Brauchle et al., 1994). In influenza-induced tissue damage, IL-1β and TNF-α are induced to be expressed in type II alveolar epithelial cells and contribute to alveolar regeneration by acting directly on alveolar epithelial cells via surface receptors and NF-κB signaling pathways (Katsura et al., 2019). In addition, mice deficient in IL-1β have been found to have an increased incidence of cysts exposed to UV light, which was shown to be associated with a reduction in skin inflammation (Kulkarni et al., 2017). Thus, the release of moderate amounts of IL-1β following the action of the danger signal ATP in gout contributes to the induction of self-resolution of inflammation in the organism. Similarly, P2X7R has a physiological function to maintain the body’s metabolic balance, and excessive inhibition interferes with normal physiological functions. Studies have found that deficiency or inhibition of P2X7R results in impaired pancreatic β-cell function, imbalance in fat distribution, decreased systemic energy expenditure and fatty acid oxidation, and increased metabolic syndrome (Glas et al., 2009; Beaucage et al., 2014; Giacovazzo et al., 2018). Therefore, moderate rather than complete inhibition of P2X7R function contributes to the timely response of the immune system and is more appropriate for disease treatment.

**FIGURE. 11:**
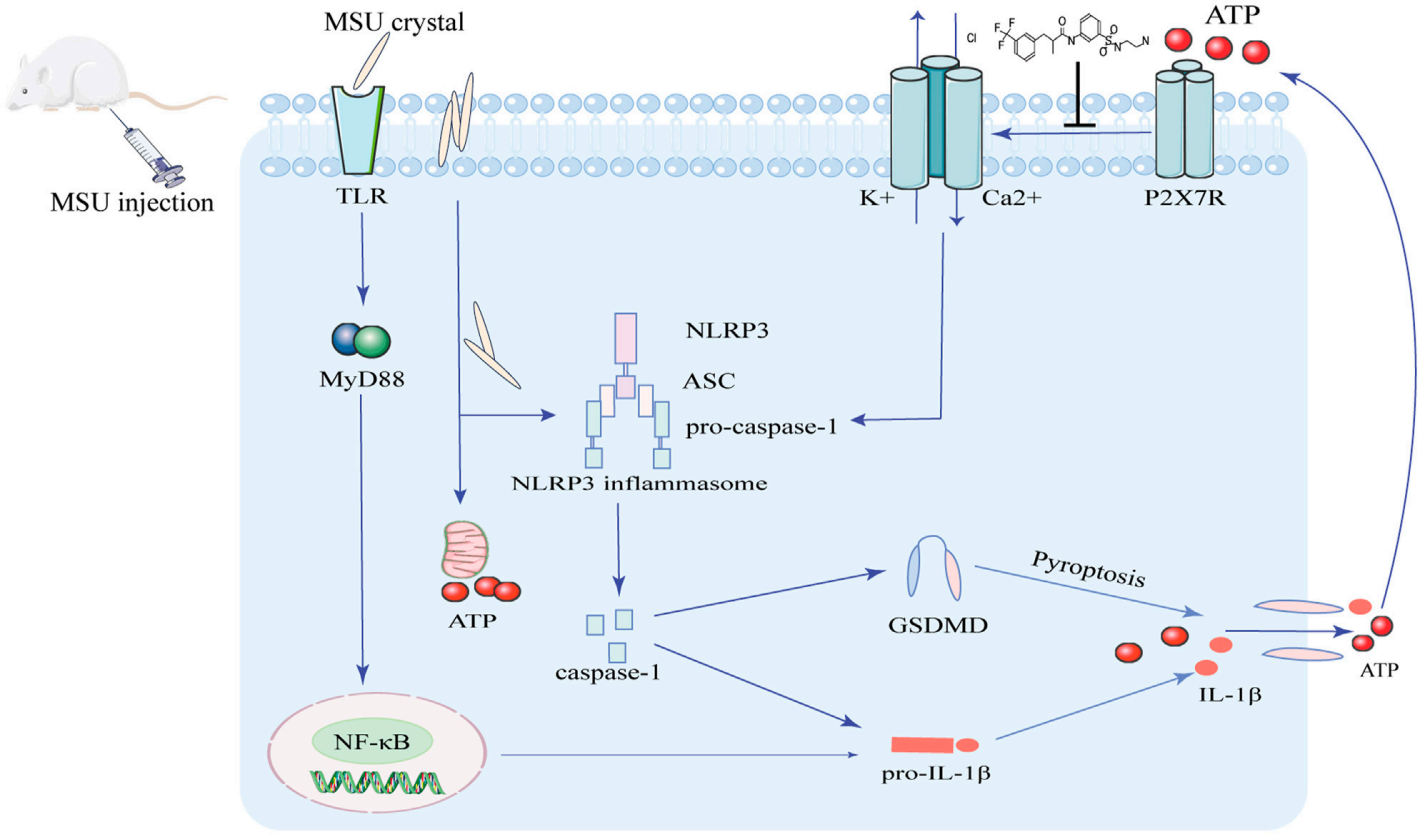
Schematic diagram of the mechanism of Z1456467176 in the treatment of MSU crystal-induced acute gouty arthritis. MSU crystal stimulation promotes ATP production, which is released through caspase-1-dependent pyroptosis. The interaction of extracellular ATP with P2X7R alters the structure of the protein and induces ion flow. Together with MSU crystals, it activates the NLRP3 inflammasome and promotes the release of sufficient IL-1β to trigger the gout inflammatory response. Z1456467176 inhibits ATP-induced channel opening and P2X7R-NLPR3-IL-1β pathway activation by remodeling the structure of P2X7R protein. Thus, acute gouty arthritis induced by MSU crystals was relieved.

In conclusion, the study identified a potential P2X7R antagonist, Z1456467176, that can inhibit ATP-induced NLRP3-caspase-1-IL-1β pathway activation by allosterically modulating P2X7R, effectively alleviating MSU crystal-induced gouty joint inflammation in rats (Figure 11). These data provide direct experimental evidence for the involvement of P2X7R in gout pathogenesis, establishing that allosteric inhibition of P2X7R is an effective strategy for gout treatment.

## Data Availability

The datasets presented in this study can be found in online repositories. The names of the repository/repositories and accession number(s) can be found in the article/Supplementary Material.
