# Supplementary material for: Z1456467176 alleviates gouty arthritis by allosterically modulating P2X7R to inhibit NLRP3 inflammasome activation
Source: Front Pharmacol. 2022 Aug 16;13:979939. doi: 10.3389/fphar.2022.979939 (PMC9424684; doi:10.3389/fphar.2022.979939)
Supplement: Supplementary file 1 [file Table1.DOCX]

Supplementary Material

# Supplementary Figure

**FIGURE. S1 IC_50_ value of Z1456467176 from the EB uptake assay.** Z1456467176 blocked ATP-induced EB uptake by HEK-293T cells overexpressing hP2X7R at concentrations ranging from 1 nM to 100 μM. Data are presented as means ± S.E.M. Statistics were analyzed using the probit analysis. n=3.

| A   | B   |
| --- | --- |

**FIGURE. S2 Z1456467176 did not affect LPS/****Nigericin-induced IL-1β secretion *in vitro.*** ELISA of IL-1β in culture supernatants of PBMCs from gout patients, treated with Z1456467176 (50 μM) for 30 min, followed by LPS (50 ng/ml) stimulation for 3 h (A) or nigericin induction for 30 min (B). Data are presented as means ± S.E.M. Statistics were analyzed using the independent samples *t*-test. n=3.

# Supplementary Table

**Table S1 Contribution of amino acid residues to binding free energy**

| **Residue** | **MM Energy** | **Polar Energy** | **APolar Energy** | **Total Energy** |
| --- | --- | --- | --- | --- |
| **GLU-55(A)** | 1.2675 | -0.0332 | 0 | 1.2341 |
| **SER-59(A)** | -5.9393 | 10.7887 | -0.4522 | 4.3988 |
| **SER-60(A)** | -2.3087 | 0.5688 | -0.1003 | -1.843 |
| **VAL-61(A)** | -1.6482 | 0.7699 | -0.1722 | -1.0515 |
| **ASN-100(A)** | -6.1908 | 8.7653 | -0.7021 | 1.8829 |
| **ASP-318(A)** | -2.2505 | 1.2059 | 0 | -1.0447 |
| **SER-59(B)** | -4.3417 | 8.4747 | -0.423 | 3.7144 |
| **VAL-61(B)** | -7.7981 | 3.7159 | -0.702 | -4.7834 |
| **HIS-62(B)** | -1.7933 | 0.2241 | -0.024 | -1.5927 |
| **THR-63(B)** | -1.0713 | -0.0763 | -0.0703 | -1.2169 |
| **LEU-97(B)** | -4.0407 | 2.21 | -0.3591 | -2.1887 |
| **ASN-100(B)** | -27.5271 | 33.353 | -2.0777 | 3.7415 |
| **ASP-318(B)** | -4.0346 | 8.9742 | -0.1482 | 4.7742 |
| **LEU-320(B)** | -3.8157 | 1.2286 | -0.2887 | -2.8758 |
| **GLU-55(C)** | 0.9505 | 0.1288 | 0 | 1.0795 |
| **GLY-99(C)** | -6.9545 | 10.2898 | -0.2655 | 3.0697 |
| **ASN-100(C)** | -5.2033 | 4.6972 | -0.5957 | -1.1109 |
| **SER-101(C)** | -3.8761 | 5.7868 | -0.1995 | 1.713 |
| **ARG-316(C)** | -1.0687 | 2.7365 | -0.257 | 1.3975 |
| **PHE-317(C)** | -0.4978 | 0.312 | 0 | -0.1854 |
| **ASP-318(C)** | -13.328 | 27.4183 | -0.3103 | 13.8036 |
| **LEU-320(C)** | -1.963 | 0.2842 | -0.2089 | -1.8878 |

The data sets related to molecular dynamics in this study can be found in the online repository. https://www.jianguoyun.com/p/DQIvL7MQmL7dChjV8soEIAA
