# Supplementary material for: Z1456467176 alleviates gouty arthritis by allosterically modulating P2X7R to inhibit NLRP3 inflammasome activation
Source: Front Pharmacol. 2022 Aug 16;13:979939. doi: 10.3389/fphar.2022.979939 (PMC9424684; doi:10.3389/fphar.2022.979939)
Supplement: Supplementary file 2 [file DataSheet1.PDF]

MaxPeak: 100.00%
Ret_Time: 0.971 min

6915548$1

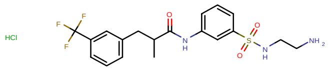

Mol Wt 465.92
Exact Mass 429.16

#	Time	Area%
1	0.971	100.00

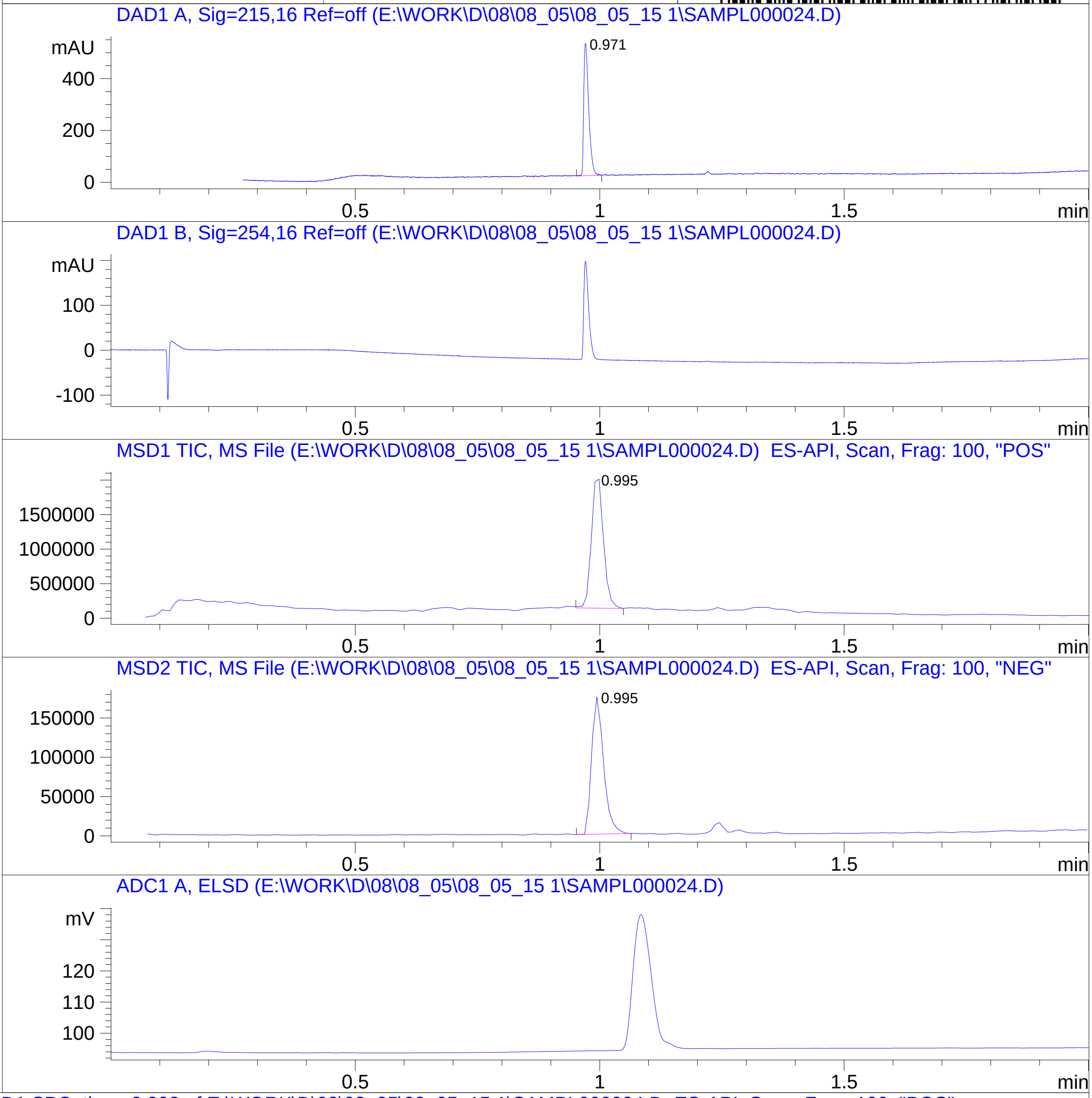

RT 0.995

RT 0.995

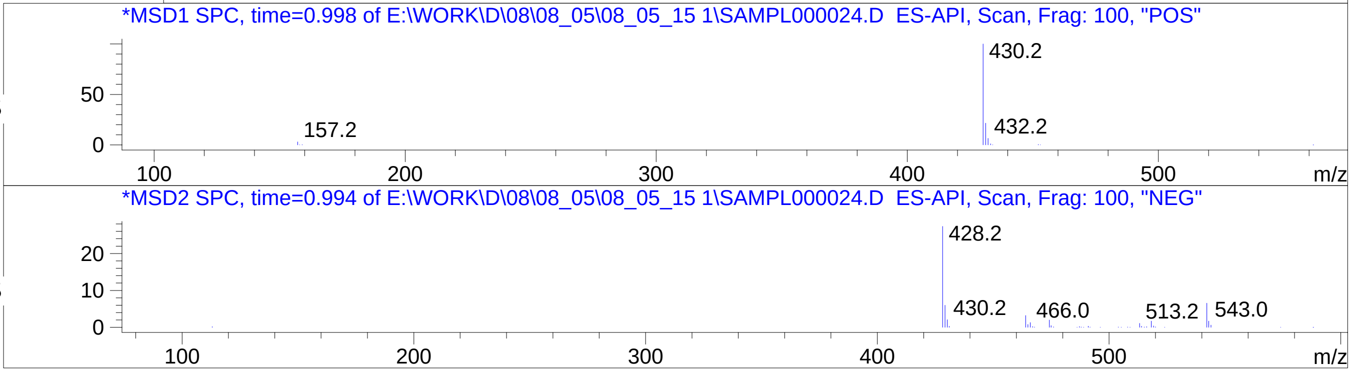

Inj.Date 8/5/2014 K P2-C-06 -5- Acq. Method C:\CHEM32\-> ->
